# Complete mitochondrial genome of *Teratoscincus przewalskii* (Reptilia, Squamata, Sphaerodactylidae) and phylogenetic analysis

**DOI:** 10.1080/23802359.2021.1987162

**Published:** 2021-10-12

**Authors:** Hui Yu, Yang Liu, Yan Liu, Junmei Yang, Siqi Li, Junhuai Bi, Ruidong Zhang

**Affiliations:** a College of Life Sciences and Technology, Inner Mongolia Normal University, Hohhot, Inner Mongolia, China; b Key Laboratory of Biodiversity conservation and Sustainable utilization for College and University of Inner Mongolia Autonomous Region, Hohhot, Inner Mongolia, China

**Keywords:** Mitochondrial genome, Squamata, Sphaerodactylidae, *Teratoscincus przewalskii*

## Abstract

The complete mitochondrial genome of the lizard, *Teratoscincus przewalskii*, which belongs to the family Sphaerodactylidae was determined based on Illumina data in this study. The result showed that the closed double-stranded circular mitogenome was 16,779 bp in total length (GenBank accession number: MW491837) with 44.07% GC. The complete mitochondrial genome consisted of 13 protein-coding genes, 22 transfer RNA genes, two ribosomal genes, and one noncoding control region. Phylogenetic analysis using mitochondrial genomes suggested that *T. przewalskii* was most closely related to its congener *T. keyserlingii*. This work provides valuable molecular information for further research on species identification and molecular evolution.

The Przesalski’s wonder gecko, *Teratoscincus przewalskii*, belonging to the subfamily Teratoscincinae (Squamata: Sphaerodactylidae), is known to be mainly distributed in Mongolia and Xinjiang, Gansu and Inner Mongolia in China (Gamble et al. 2007, 2011, 2012; Pyron et al. 2013; Nazarov et al. 2017). This species mostly lives in arid Gobi gravel sand, fixed dunes, semi-quicksand zones and Gobi Desert near reclaimed land. In this paper, we described the characteristics of the mitochondrial genome of *T. przewalskii* and discussed the phylogenetic relationships among Gekkota species, in order to provide a basis for further studies on interspecific taxonomy and phylogenetic relationships of these taxa.

*Teratoscincus przewalskii* was collected in Ejin Banner, Alxa League, Inner Mongolia, China in September 2020 (42.23 N, 101.31E) and was deposited in the laboratory of the College of Life Sciences and Technology of Inner Mongolia Normal University, Hohhot, China (http://bio.imnu.edu.cn/, Hui Yu, yuhuilbc@163.com). The muscular tissues were obtained and preserved in 95% ethanol. Total genomic DNA was extracted using the Qiagen Blood & Tissue Kit (QIAGEN, Hilden, Germany). Genomic DNA samples after testing qualified, with the method of mechanical interrupt (ultrasonic) DNA fragmentation, then end of fragmented DNA fragment purification, repair, and 3′ end and A sequencing, connection joints, and then selected fragment by agarose gel electrophoresis and sequencing library was formed by PCR amplification. After the library is built, the library quality inspection should be carried out. The qualified libraries were sequenced using Illumina Novaseq platform. The mitogenome was assembled by SPAdes v3.10.1 software (http://cab.spbu.ru/software/spades/) using *Teratoscincus roborowskii* (GenBank accession number: MW491837) as reference (Bankevich et al. 2012). The complete mitochondrial genome was annotated using Mitos2 (http://mitos2.bioinf.uni-leipzig.de), The genome sequence data that support the findings of this study are openly available in GenBank of NCBI at [https://www.ncbi.nlm.nih.gov] (https://www.ncbi.nlm.nih.gov/) under the accession no. MW491837.

The complete mitochondrial genome of *T. przewalskii* was a circular molecule with 16,779 bp in total length (GenBank accession number: MW491837) and contained 13 protein-coding genes (PCGs), 22 transfer RNA (tRNA) genes, two ribosomal RNA (rRNA) genes, and one noncoding control region. The overall nucleotides composition was 30.90% A, 25.03% T, 13.78% G, and 30.29% C, which showed a bias toward A + T (55.93%). These were consistent with values found in other vertebrate species (Böhme et al. 2007; Li et al. 2016). Among the 13 PCGs, the common start codons (ATG and GTG) could be assigned as the start codon for most of PCGs and ND2, ND3 begin with ATA, only ND1 begins with ATC. The stop codons of the PCGs were TAA (ATP8, ATP6, ND4L, ND4, ND6), TAG (ND2, ND5, Cytb), TA (ND1, ND3), AGG (COI), T (COII, COIII). The size of the 22 tRNA genes ranged from 66 (tRNA^Cys^, tRNA^Val^) to 75 (tRNA^Leu^) nucleotides. All tRNAs were foldable, with a typical clover structure, and their anticodon was exactly the same as the vertebrate tRNAs sequenced (Yan et al. 2009, Li et al. 2016, Yang et al. 2021). Two rRNA genes, 12S rRNA (948 bp) and 16S rRNA genes (1545 bp) were located between tRNA^Phe^ and tRNA^Leu^, separated by tRNA^Val^. The control region (1369 bp) was located between tRNA^Pro^ and tRNA^Phe^.

Based on the complete mitochondrial genome of *T. przewalskii* and other 22 species of Gekkota, a phylogenetic tree was constructed using Maximum-likelihood (ML) method on RAxML v8.2.10 software (https://cme.h-its.org/exelixis/software.html）with 1000 bootstrap replicates (Stamatakis 2014). The result showed that *T. przewalskii* was most closely related to its congener *T. keyserlingii* and rooted with the other Gekkonidae species (Han et al. 2004; Macey et al. 2005; Harris and Rato 2008; Nazarov et al. 2017) (Figure 1). This mitochondrial genome provides valuable molecular information for further research on species identification and molecular evolution.

**Figure 1.**
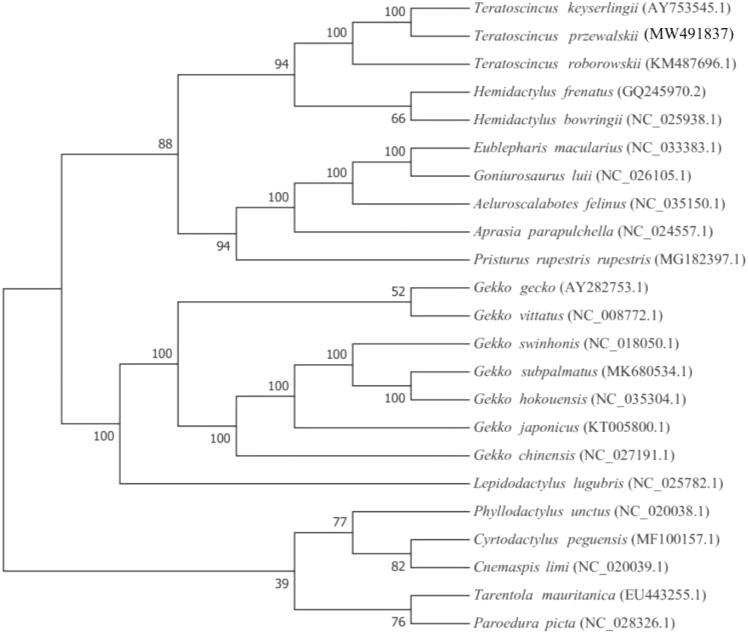
Phylogenetic position of *T. przewalskii* based on a comparison with the complete mitochondrial genome sequences of 22 other Gekkota species. The analysis was performed using RAxML v8.2.10 software. The accession number for each species is indicated after the scientific name.

The inferred position of *T. przewalskii* herein was contradictory to previous and most recent publications (Macey et al. 1999, 2005; Tamar et al. 2021), which strongly support the sister relationship bewteen *T. przewalskii* and *T. roborowskii*. Whereas the previous publications used sequence lengths of NAD1 to COS1, we used the full sequence. This is the main reason for the difference in our analysis results. We still need more data in the future to verify the results.

## Data Availability

The data that support the findings of this study are openly available in GenBank of NCBI at https://www.ncbi.nlm.nih.gov, reference number MW491837.
